# Skeletal muscle reprogramming in peripheral nerve injury: mechanisms, therapeutic roles, and complication management

**DOI:** 10.3389/ebm.2026.10835

**Published:** 2026-03-02

**Authors:** Fuqiang Long, Xiaoru Pan, Anxin He, Xinlu Wang, Zairong Wei, Shaoying Gao

**Affiliations:** 1 Department of Burns and Plastic Surgery, Affiliated Hospital of Zunyi Medical University, Zunyi, China; 2 The 2011 Collaborative Innovation Center of Tissue Damage Repair and Regeneration Medicine, Affiliated Hospital of Zunyi Medical University, Zunyi, China; 3 The Collaborative Innovation Center of Tissue Damage Repair and Regeneration Medicine, Zunyi Medical University, Zunyi, China

**Keywords:** skeletal muscle reprogramming, peripheral nerve injury, therapeutic strategies, complications, regeneration medicine

## Abstract

Peripheral nerve injury (PNI) presents a significant clinical challenge, frequently leading to long-term neuromuscular dysfunction, muscle atrophy, fibrosis, and chronic pain. Traditional repair strategies, including microsurgical reconnection and neurotrophic support, often yield limited functional recovery, especially in cases of delayed or incomplete reinnervation. In this context, skeletal muscle reprogramming—defined as the intentional modulation of cellular fate, function, or metabolic state in muscle-resident cells—has emerged as a promising strategy to enhance regenerative outcomes. This process involves transcriptional, epigenetic, and metabolic interventions targeting myogenic progenitors, fibro-adipogenic progenitors (FAPs), satellite cells (MuSCs), and the broader muscle microenvironment. Recent studies demonstrate that reprogramming strategies can mitigate denervation-induced muscle atrophy, delay fibrotic remodeling, promote neuromuscular junction (NMJ) reconstruction, and even stimulate endogenous nerve regrowth via retrograde signaling. Mechanistic insights have uncovered pivotal roles for signaling pathways such as Wnt/β-catenin, TGF-β, Notch, and HDAC-regulated chromatin dynamics. Furthermore, innovations in small molecule cocktails, CRISPR-based transcriptional reactivation, and metabolic rewiring have expanded the therapeutic toolkit for muscle preservation and regeneration. This review comprehensively examines the molecular mechanisms, therapeutic roles, and translational challenges of skeletal muscle reprogramming in the context of PNI. We explore how muscle-targeted interventions can address complications of denervation, improve the efficacy of nerve repair, and offer a synergistic axis of regeneration when integrated with nerve-centric strategies. Finally, we identify key knowledge gaps and outline future research directions required to translate reprogramming-based therapies into clinical practice.

## Impact statement

This work introduces skeletal muscle reprogramming as a transformative approach for managing peripheral nerve injuries (PNIs), offering a novel perspective by targeting muscle regeneration, rather than solely focusing on nerve repair. The insights provided here bridge a critical gap in current therapeutic strategies, addressing the complications of denervation-induced muscle atrophy, fibrosis, and chronic pain.

The article advances the field by synthesizing the latest molecular, epigenetic, and metabolic approaches to skeletal muscle reprogramming, particularly in PNI contexts. It highlights how muscle-targeted therapies, integrated with nerve-centered strategies, can improve regenerative outcomes and functional recovery. This work presents new insights into the mechanisms underlying muscle reprogramming, such as the role of signaling pathways (e.g., Wnt/β-catenin, TGF-β) and small molecule cocktails. It introduces the potential for retrograde signaling to stimulate nerve regrowth and neuromuscular junction (NMJ) reconstruction, expanding the therapeutic toolkit for PNI recovery.

The new information shifts the paradigm from treating muscle and nerve damage separately to adopting a synergistic approach. By focusing on muscle reprogramming, this work may lead to more effective, holistic treatments for PNI, particularly for cases with poor recovery due to prolonged or incomplete reinnervation.

## Introduction

Peripheral nerve injuries (PNIs) affect millions globally and are commonly associated with trauma, surgery, or chronic compression syndromes [1]. Despite advances in microsurgical techniques and neurotrophic factor delivery, the functional recovery following PNI remains suboptimal—particularly in proximal or delayed repairs—largely due to irreversible changes in the denervated skeletal muscle [2]. Denervation triggers a cascade of degenerative events in muscle tissue, including fiber atrophy, fibrosis, mitochondrial dysfunction, and motor endplate destabilization, all of which significantly compromise reinnervation and functional restitution [3, 4]. Historically, therapeutic interventions for PNI have centered on promoting axonal regrowth and guiding nerve regeneration. However, emerging evidence underscores the critical importance of preserving or reactivating the denervated target tissue—primarily skeletal muscle—as an equally essential component of successful nerve repair. Indeed, the muscle environment plays an active role in modulating neuromuscular regeneration [5–7], through retrograde signaling, extracellular matrix remodeling, and trophic support for regenerating axons [6, 8, 9]. Skeletal muscle reprogramming represents a paradigm shift in the treatment of PNI and its complications [10]. Rather than focusing solely on neuronal repair, reprogramming strategies aim to convert, preserve, or rejuvenate muscle-resident cells—such as satellite cells, fibro-adipogenic progenitors (FAPs), and even mature myofibers—via targeted molecular interventions [11, 12]. This approach not only prevents muscle degeneration but may also transform the injured muscle into a more permissive and instructive environment for axonal regeneration. In this review, we systematically explore the concept of skeletal muscle reprogramming in the context of peripheral nerve injury. We provide an overview of its underlying cellular and molecular mechanisms, examine current experimental and translational strategies, and evaluate its potential to mitigate PNI-associated complications such as muscle fibrosis and chronic pain. Additionally, we highlight the intersection between muscle biology and nerve regeneration, proposing skeletal muscle not merely as a passive target but as an active participant in neuromuscular repair.

## Pathophysiology of peripheral nerve injury and complications

**FIGURE 1:**
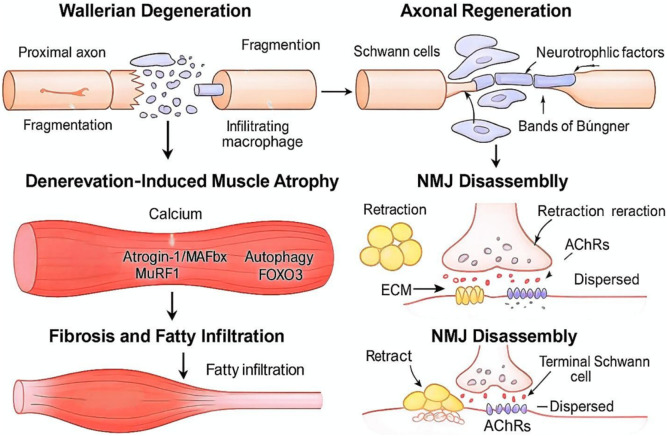
Pathophysiology of Peripheral Nerve Injury and Muscle Degeneration. This figure illustrates the sequential pathological cascade triggered by peripheral nerve injury, encompassing nervous system changes and target muscle degeneration. The section on Wallerian degeneration depicts the fragmentation of the distal axon and the subsequent clearance of debris by Schwann cells and macrophages. Denervation-induced muscle atrophy is highlighted through the activation of proteolytic and autophagic pathways, such as those involving Atrogin-1, MuRF1, and FOXO3, which lead to a rapid loss of muscle mass. Furthermore, the figure reveals the aberrant activation of fibro-adipogenic progenitors (FAPs) and their differentiation into fibroblastic and adipogenic lineages, ultimately resulting in tissue fibrosis and fatty infiltration.

Peripheral nerve injury (PNI) initiates a complex and time-sensitive cascade of degenerative and regenerative events, impacting both the nervous system and its target organs, primarily skeletal muscle. Understanding the cellular and molecular basis of PNI is critical to identifying the therapeutic window for skeletal muscle reprogramming and its role in functional recovery (Figure 1).

### Classification and etiology of PNI

Peripheral nerve injuries (PNIs) are stratified by severity according to Seddon’s and Sunderland’s systems. Seddon’s scheme distinguishes three types—neurapraxia (transient conduction block), axonotmesis (axon disruption with intact connective sheaths), and neurotmesis (complete nerve transection)—while Sunderland refines this into five grades, specifying the extent of damage to axons, endoneurium, perineurium, and epineurium [13, 14]. Etiologies range from blunt trauma and sharp lacerations to iatrogenic injury during surgery [15], tumor compression, and inflammatory neuropathies. The potential for regeneration diminishes as injury severity increases and as the interval to reinnervation lengthens; hence, prompt diagnosis and intervention are critical to maintain muscle responsiveness and prevent irreversible atrophic changes [16].

### Wallerian degeneration and axonal regeneration

Following axonal transection, Wallerian degeneration ensues within hours: the distal axon and myelin sheath fragment and are cleared by Schwann cells and infiltrating macrophages [17]. Concurrently, Schwann cells adopt a reparative phenotype, upregulating neurotrophic factors such as NGF, GDNF, and BDNF, and aligning into Bands of Büngner that channel regrowing axonal sprouts [18]. Despite this orchestrated response, axonal regrowth proceeds slowly—typically 1–3 mm per day—and often fails to bridge long gaps, especially in proximal injuries or when surgery is delayed [19]. Such delays exacerbate target muscle atrophy and highlight the necessity of preserving the muscle niche to optimize reinnervation [20].

### Denervation-induced skeletal muscle atrophy

Denervated muscle fibers rapidly undergo atrophy driven by disrupted calcium homeostasis, oxidative stress, and activation of proteolytic systems such as the ubiquitin–proteasome pathway (via atrogin-1/MAFbx and MuRF1) [21]. Autophagy–lysosome activity is also upregulated early after denervation, facilitating removal of damaged organelles but becoming insufficient at later stages of atrophy [22]. FOXO3-mediated transcriptional upregulation of atrophy-related genes further amplifies catabolic signaling [23]. Concurrently, the IGF-1/Akt/mTOR axis is downregulated, reducing anabolic drive and protein synthesis [24]. Loss of neural stimuli also triggers sarcomere disassembly, shifts fiber-type composition toward fast-twitch phenotypes, and exacerbates mitochondrial dysfunction [25].

### Fibrosis and fatty infiltration

With prolonged denervation, myofibers are progressively replaced by fibrotic and adipose tissue [26]. Fibro‐adipogenic progenitors (FAPs), normally transiently activated after injury, proliferate unchecked in the absence of reinnervation, driving extracellular matrix (ECM) deposition through TGF-β signaling [27]. This process is exacerbated by a dysregulated balance of matrix metalloproteinases and their inhibitors (MMPs and TIMPs), leading to ECM stiffening that impedes myogenic repair and creates a physical barrier to regenerating axons [28]. Simultaneously, ectopic lipid accumulation from FAPadipogenic differentiation further degrades muscle architecture and metabolic homeostasis [29].

### Neuromuscular junction (NMJ) disassembly

The NMJ, comprising the presynaptic motor terminal, the postsynaptic muscle membrane with clustered acetylcholine receptors (AChRs), and terminal Schwann cells, deteriorates rapidly after denervation [30]. AChR clusters disperse and synaptic folds flatten as presynaptic terminals retract; myonuclei drift from the subsynaptic region [31]. Initially, terminal Schwann cells extend processes that guide regenerating axons, but prolonged denervation leads to Schwann cell apoptosis, undermining reinnervation fidelity [32]. Restoring NMJ architecture thus requires coordinated nerve and muscle repair strategies [33].

### Pain and sensory complications

PNI frequently precipitates neuropathic pain, marked by ectopic neuronal firing and central sensitization [34]. Denervated muscle contributes to pain through increased proinflammatory cytokines (e.g., IL-6, TNF-α), altered metabolic signaling, and heightened peripheral nociceptor sensitivity [35]. Effective management of sensory symptoms therefore demands both neurocentric interventions and strategies that modulate the denervated muscle environment, further supporting a role for muscle reprogramming in comprehensive PNI management [36].

## Concept and classification of skeletal muscle reprogramming

### Definition and scope

**S**keletal muscle reprogramming encompasses deliberate interventions that alter the fate or functional phenotype of muscle-resident or stromal cells to restore or enhance regeneration after injury. Unlike classical myogenesis, which relies solely on activation of endogenous satellite cells, reprogramming employs transcriptional, epigenetic, and metabolic cues to override impaired regenerative programs [37–39]. By reinforcing myogenic commitment in senescent MuSCs [40], converting fibro-adipogenic progenitors into myogenic-like cells [26, 41], or modulating the niche to support reinnervation, angiogenesis, and antifibrotic remodeling [42], this approach extends beyond mere muscle repair. In the setting of peripheral nerve injury (PNI), where denervated muscle rapidly atrophies and undergoes fibrosis and synaptic loss, skeletal muscle reprogramming serves as a critical adjunct to nerve-centered therapies.

### Key target cell populations

Muscle satellite cells (MuSCs), marked by Pax7, are the principal drivers of myofiber regeneration. Under chronic denervation or aging, MuSCs enter senescence or apoptosis, depleting the progenitor pool [43]. Reprogramming strategies restore MuSC function bysuppressing oxidative stress [44], inhibiting NF-κB–mediated inflammatory suppression [45], and remodeling histone marks (H3K27ac) to revive stemness [46]. Fibro-adipogenic progenitors (FAPs) normally support acute repair but drive fibrosis and fatty infiltration under chronic insult. Redirecting FAP fate through TGF-β/Smad blockade and IL-4/STAT6 activation promotes secretion of pro-myogenic factors such as follistatin while suppressing adipogenesis [8, 47]. Moreover, epigenetic remodeling ofFAP-derived extracellular vesicles with miR-206/133 cargo enhances MuSC activity and niche rewiring via M2 macrophage polarization or GDNF-mediated innervation further restrains FAP dysregulation. Fibroblasts and myofibroblasts, which contribute to ECM deposition and scarring after PNI, can be transdifferentiated into induced myogenic progenitor cells (iMPCs) using MyoD overexpression, small-molecule cocktails, and HDAC inhibition; yet this conversion is limited by TGF-β–driven epigenetic barriers, matrix stiffness, and inflammation [48]. Although not direct reprogramming targets, immune and vascular cells are reshaped by muscle-centric interventions, with M2 macrophages and neovessels forming supportive niches [49]. Lastly, mature myofibers exhibit metabolic and structural plasticity: interventions such as AMPK activation or NAD^+^ supplementation stimulate mitochondrial biogenesis and shift fiber-type profiles, creating a substrate more conducive to reinnervation [50].

### Inductive modalities

Transcriptional reprogramming via overexpression of myogenic factors like MyoD or Pax7 can restart myogenesis in fibroblasts or senescent MuSCs; however, the balance between these factors is critical, as Pax7 overexpression may inhibit MyoD-driven differentiation [51]. Epigenetic modulation uses HDAC inhibitors and DNA methylation inhibitors such as 5-azacytidine to relax chromatin, facilitating activation of muscle-specific genes [52]. Chemical cocktails that recapitulate developmental signals—combining forskolin, CHIR99021, FGF2, and IGF-1—activate Wnt/β-catenin and PI3K/AKT pathways to induce reprogramming without genetic manipulation [53]. CRISPR-mediated activation (CRISPRa) employs dCas9 fused to transcriptional activators to upregulate endogenous Myod1 and Myf5 with spatial and temporal precision [54]. Finally, metabolic and mechanical cues such as hypoxia-induced ROS, ECM stiffness, and cyclic stretch modulate mechanotransduction pathways (e.g., YAP/TAZ) to influence cell fate decisions [55].

### Classification of reprogramming strategies

Reprogramming approaches can be categorized mechanistically into four classes (Table 1). Transcriptional reprogramming involves forced expression or activation of myogenic transcription factors, exemplified by MyoD-driven conversion of fibroblasts [56]. Epigenetic reprogramming modulates chromatin accessibility via HDAC inhibitors, DNA demethylases like 5-aza, or CRISPRa/dCas9 tethered to epigenetic effectors [57, 58]. Metabolic reprogramming alters bioenergetic states through AMPK activation or NAD^+^ supplementation, enhancing mitochondrial function and oxidative capacity [50]. Pharmacological reprogramming utilizes cytokines and small molecules—such as IGF-1, Wnt agonists, and TGF-β inhibitors—to shift cell fate trajectories [53, 59]. This framework informs tailored therapy design for specific PNI-related pathologies, whether combating atrophy, fibrosis, or NMJ instability.

**TABLE 1:** Mechanistic Classification of skeletal muscle reprogramming approaches.

Strategy	Mechanism	Advantages	Limitations	References
Transcriptional	Forced expression or activation of myogenic transcription factors (e.g., MyoD, Pax7)	High efficiency in direct lineage conversion and myogenic commitment Pt	Potential risk of oncogenic transformation and formation of immature or unstable myofibers [s]	[58]
Epigenetic Mc	Modulation of chromatin accessibility via HDAC inhibitors, DNA demethylases, or CRISPRa-effectors Fs	Facilitates activation of endogenous muscle-specific genes without introducing exogenous sequences Rs	Risk of unintended global epigenetic shifts and potential for off-target gene activation [n]	[59, 60]
Metabolic Ac	Alteration of cellular bioenergetics to boost mitochondrial function (e.g., AMPK agonists, NAD+) m	Mitochondrial function (e.g., AMPK agonists, NAD+) high clinical feasibility and safety; addresses the metabolic decline associated with denervation n	Primarily addresses bioenergetics; may be insufficient to drive full structural regeneration in chronic injury [y]	[61]
Pharmacological Ul	Use of cytokines and small molecules to influence signaling pathways (e.g., IGF-1, Wnt agonists)	Highly translatable and compatible with injectable delivery systems or smart biomaterials ss	Short molecular half-life requires sustained release systems to maintain therapeutic levels [s]	[62, 63, 131]

This table systematically classifies skeletal muscle reprogramming strategies based on their molecular intervention levels and includes a critical evaluation of their advantages and limitations to address reviewer recommendations. Transcriptional reprogramming utilizes the forced expression of myogenic regulatory factors such as MyoD to achieve high-efficiency lineage conversion, though it carries risks of oncogenic transformation and the formation of immature myofibers. Epigenetic reprogramming modulates chromatin accessibility to activate endogenous genes without introducing exogenous sequences, but it must be carefully monitored for off-target modifications. Metabolic reprogramming targets the bioenergetic decline in denervated muscle, offering high clinical safety and feasibility, yet it may be insufficient to drive full structural regeneration independently. Pharmacological reprogramming employs small molecules to flexibly modulate signaling pathways with high local controllability, but its efficacy is often limited by the short half-life of these molecules.

### Combinatorial and sequential modalities

To maximize efficacy, emerging studies advocate for integrated, staged interventions. In a murine denervation model, sequential administration of CHIR99021 and HDAC inhibitors followed by MyoD-CRISPRa achieved superior myofiber regeneration compared to any single modality, illustrating the synergism of metabolic priming, epigenetic opening, and transcriptional activation [60]. Such combinatorial regimens may be delivered via smart biomaterials that release agents in response to enzymatic or pH changes in the injured niche, providing temporal control aligned with endogenous repair phases [61, 62].

## Molecular mechanisms of skeletal muscle reprogramming

**FIGURE 2:**
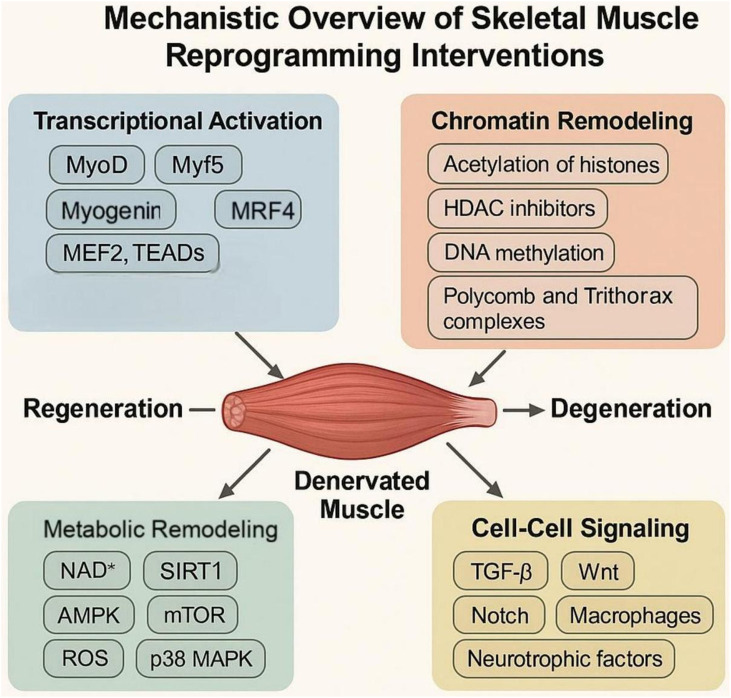
Mechanistic Overview of Skeletal Muscle Reprogramming Interventions. This figure provides a comprehensive mechanistic framework for reprogramming interventions in the context of peripheral nerve injury. It details the synergistic interplay between transcriptional activation, chromatin remodeling, metabolic reconfiguration, and intercellular signaling pathways such as Wnt, Notch, and TGF-β. These reprogramming strategies do not only target mature myofibers to improve contractile performance but also reshape the regenerative microenvironment by modulating extracellular matrix (ECM) components and inducing M2 macrophage polarization, aiming to transform the degenerated muscle niche into a supportive environment for axonal regeneration.

Skeletal muscle reprogramming involves a complex interplay of transcriptional activation, chromatin remodeling, metabolic reconfiguration, and cell–cell signaling. These molecular mechanisms determine whether denervated muscle undergoes regeneration, fibrosis, or irreversible degeneration. Understanding these processes provides a basis for the rational design of reprogramming therapies in the context of peripheral nerve injury (PNI) (Figure 2).

### Transcriptional control of myogenic identity

Transcription factors (TFs) are primary regulators of cellular identity and lineage reprogramming. In skeletal muscle, the core myogenic regulatory factors (MRFs)— MyoD, Myf5, Myogenin, and MRF4—coordinate muscle-specific gene expression and lineage commitment. MyoD, in particular, can convert non-myogenic cells, such as fibroblasts, into myoblast-like cells by binding to E-box elements and recruiting epigenetic coactivators [63]. Pax7 is another key TF that maintains the self-renewal and quiescence of satellite cells; its acetylation status regulates muscle stem cell function and differentiation potential [64]. TF networks are further modulated by context-specific cofactors such as MEF2, TEADs, and YAP/TAZ, which integrate developmental cues and mechanical signals to fine-tune myogenic outputs [65, 66].

### Epigenetic remodeling and chromatin dynamics

Epigenetic modifications govern the accessibility of myogenic loci, influencing a cell’s responsiveness to reprogramming signals. Histone acetylation, such as H3K9ac mediated by p300/CBP, facilitates open chromatin at myogenic enhancers, while histone deacetylation by HDACs represses myogenic genes. HDAC inhibitors, like trichostatin A, have been shown to enhance MyoD-induced conversion efficiency [67]. DNA methylation also plays a crucial role; hypermethylation of muscle-specific promoters (e.g., Myf5) can block myogenic differentiation, whereas inhibitors like 5-azacytidine demethylate these regions and restore transcriptional competency [68]. Chromatin regulators, such as the Polycomb and Trithorax complexes, dynamically repress or activate gene loci. EZH2, a methyltransferase in PRC2, represses Pax7 and MyoD; its inhibition reactivates satellite cell proliferation in aged or denervated muscle [69]. CRISPRa/i systems, employing engineered dCas9-fused activators or repressors, offer locus-specific epigenetic modulation, enabling fine control over endogenous gene expression without genome editing [69].

### Metabolic regulation and mitochondrial rewiring

Muscle regeneration and cell fate decisions are tightly coupled to metabolic states. Oxidative metabolism favors MuSC quiescence and self-renewal, whereas glycolytic flux supports proliferation and differentiation [70]. Denervation leads to mitochondrial fragmentation and oxidative stress, impairing regenerative potential [71]. Boosting NAD^+^ levels through nicotinamide riboside or SIRT1 activation improves mitochondrial function and enhances myogenic capacity in aging and injury [72]. The AMPK and mTOR pathways are also pivotal; AMPK promotes autophagy and metabolic resilience, while mTORC1 drives protein synthesis and muscle hypertrophy [73, 74]. Their balanced modulation is essential in reprogramming strategies. Reactive oxygen species (ROS) play dual roles; while excessive ROS is damaging, moderate levels act as signaling molecules to stimulate regeneration via p38 MAPK and NRF2 pathways [75].

### Crosstalk with the extracellular niche

The extracellular matrix (ECM), vasculature, and immune system shape the reprogramming landscape via paracrine and mechanical cues. TGF-β signaling is a central inhibitor of regeneration, promoting FAP differentiation into fibrotic and adipogenic lineages; its blockade with agents such as losartan or TGF-β–neutralizing antibodies reduces fibrosis and enhances muscle regeneration in injury models [76, 77]. The Wnt/β-catenin pathway plays a dual role: canonical Wnt signaling promotes MuSC activation, whereas non-canonical ligands (e.g., Wnt5a) can impair myogenic reprogramming by inducing pro-inflammatory cascades [78]. Notch signaling maintains satellite cell quiescence and is essential during early regeneration; modulating Notch–Delta interactions (for example, via γ-secretase inhibitors or Delta-like ligand mimetics) improves functional recovery after PNI [79]. Finally, macrophage polarization from M1 to M2 phenotypes supports regenerative myogenesis—delivery of IL-4 or IL-10 to injured muscle skews macrophages toward an M2 profile, creating a pro-regenerative microenvironment that synergizes with reprogramming cues [80].

### Synaptic and neurotrophic interactions

Recent findings suggest that reprogrammed muscle can exert retrograde effects on the regenerating nerve. Muscle‐restricted overexpression of neurotrophic factors—including IGF-1, BDNF and GDNF—enhances neuronal survival and axonal elongation in models of peripheral nerve injury [81]. Myogenic reprogramming also restores the expression of key synaptic organizers, such as Rapsyn, Agrin and MuSK, which are essential for neuromuscular junction integrity [82]. Additionally, axon‐guidance molecules—including Netrin-1, Semaphorins and Slit-Robo ligands—are modulated in reprogrammed muscle, influencing reinnervation fidelity and guiding regenerating axons to their targets [83].

### Integration and therapeutic implications

Integrating these molecular mechanisms provides a comprehensive framework for developing targeted interventions in peripheral nerve repair. Combinatorial regimens that couple transcriptional activation with epigenetic modulation and metabolic priming— delivered via advanced vector systems or smart biomaterials—have demonstrated synergistic restoration of muscle mass and function in preclinical models [10]. However, clinical translation must overcome challenges in delivery, including achieving tissue-specific targeting, minimizing immunogenicity, and avoiding off-target effects inherent to viral vectors and genome editors [84, 85]. Furthermore, patient-specific variables such as age-related declines in MuSC responsiveness and heterogeneity in injury severity necessitate personalized dosing and modality selection to optimize therapeutic outcomes [86].

## Therapeutic applications of muscle reprogramming in peripheral nerve injury

Skeletal muscle reprogramming is a new way to treat peripheral nerve injury (PNI). It helps in three main ways: keeping muscles healthy, creating a better environment for healing, and improving the connection between nerves and muscles. Traditional treatments focus on the nerves, but this new approach works directly on the muscles that have lost their nerve connections. It makes the muscles not just something that nerves connect to, but an active part of the healing process.

### Preservation of muscle mass and prevention of atrophy

One of the earliest and most severe consequences of PNI is muscle atrophy, driven by a disruption in neuromuscular signaling and sustained inactivity. Prompt initiation of reprogramming strategies is essential, as delayed intervention may lead to irreversible myofiber degeneration. By reactivating myogenic programs through overexpression of transcription factors such as MyoD and Pax7, satellite cells can be re-engaged to promote tissue regeneration even in non-myogenic compartments [87, 88]. In parallel, reprogramming approaches that inhibit catabolic pathways—including FOXO3, atrogin-1, and MuRF1— have been shown to effectively reduce proteolysis, thereby preserving muscle mass [89]. Enhancing mitochondrial function through NAD^+^ supplementation and AMPK activation further supports oxidative metabolism and delays disuse atrophy [90, 91]. Preclinical studies in sciatic nerve transection models treated with small molecule cocktails have demonstrated sustained preservation of fiber cross-sectional area and contractile protein expression, even after prolonged denervation [92].

### Anti-fibrotic and anti-adipogenic remodeling

Following chronic denervation, skeletal muscle undergoes fibrotic and adipogenic remodeling, compromising both tissue function and its capacity for reinnervation [93]. Fibro-adipogenic progenitors (FAPs), which initially support regeneration, become pathological if not properly regulated. Reprogramming during the early post-injury period takes advantage of a critical window in which FAPs remain plastic and responsive [94]. Targeted inhibition of TGF-β signaling in FAPs has been shown to redirect their differentiation toward a quiescent or pro-myogenic state, thereby mitigating fibrotic scarring [95]. Simultaneously, pharmacological agents that normalize extracellular matrix composition—such as inhibitors of collagen synthesis or enhancers of matrix metalloproteinase activity—contribute to structural recovery and tissue elasticity [11]. The use ofWnt agonists and HDAC inhibitors further blocks adipogenic transcription factors like PPARγ and C/EBPα, limiting ectopic fat deposition [96]. Collectively, these effects preserve structural integrity and create a more permissive environment for axonal regrowth and synapse formation [97].

### Enhancement of neuromuscular junction (NMJ)

**FIGURE 3:**
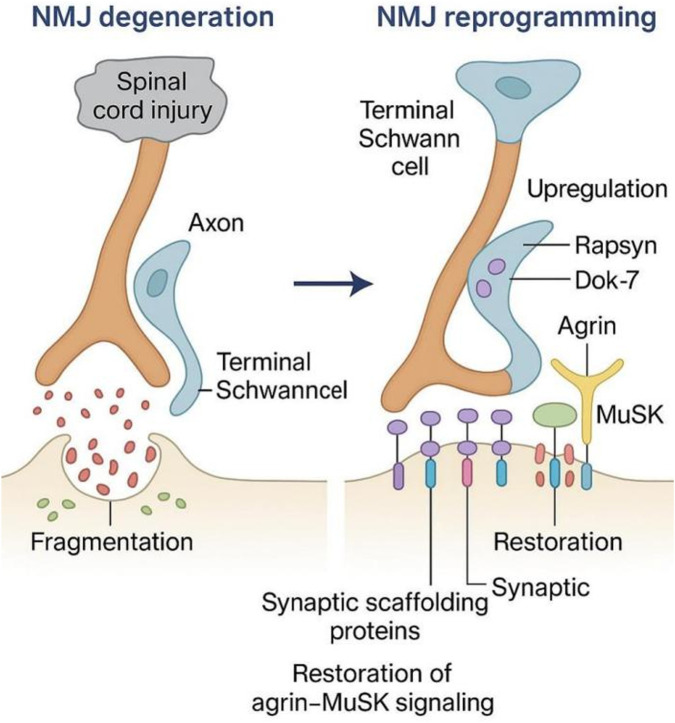
Neuromuscular Junction (NMJ) Disassembly and Reformation. This figure contrasts the disassembly of the neuromuscular junction (NMJ) following peripheral nerve injury with the process of precise reconstruction induced by muscle reprogramming. The left panel depicts the dispersal of acetylcholine receptor (AChR) clusters and the degradation of the postsynaptic membrane following denervation. The right panel illustrates the restoration of synaptic scaffolding proteins, such as Rapsyn and Dok-7, and the reactivation of the Agrin-MuSK signaling axis under reprogramming interventions. These mechanisms collectively promote functional synapse reformation and generate retrograde signals through the secretion of neurotrophic factors, such as IGF-1, BDNF, and GDNF, which enhance neuronal survival and guide regenerating axons back to their original target territories.

Following spinal cord injury, the neuromuscular junction (NMJ) is particularly vulnerable to denervation, leading to rapid disassembly characterized by fragmentation of acetylcholine receptor (AChR) clusters and degeneration of synaptic architecture [98]. However, reprogramming interventions, if implemented early—ideally within days of injury—can restore NMJ integrity [99]. This restoration involves the upregulation of synaptic scaffolding proteins such as Rapsyn and Dok-7 [100, 101], which stabilize AChR distribution across the postsynaptic membrane. Additionally, the restoration of agrin- MuSK signaling is crucial for accurate synaptogenesis, enabling regenerating axons to reform functional NMJs [102]. Furthermore, reprogramming indirectly benefits terminal Schwann cells, whose reactivation is essential for guiding axons back to their target zones [98]. Improved NMJ integrity, as demonstrated in multiple animal models, corresponds with better functional outcomes, including enhanced muscle responsiveness and coordination (Figure 3) [103].

### Promotion of retrograde signaling and axonal regeneration

While axonal regeneration has been primarily attributed to neuron-intrinsic mechanisms, recent studies demonstrate that reprogrammed muscle actively participates via retrograd signaling [11]. Muscle fibers and satellite cells subjected to reprogramming secrete neurotrophic myokines—including IGF-1, CNTF, and BDNF—which collectively enhance neuronal survival and axonal elongation [11, 104, 105]. In addition, muscle-derived axon guidance molecules such as netrin-1, semaphorins, and ephrin ligands orchestrate axonal pathfinding, ensuring precise reinnervation of target muscle territories [106]. The release of synaptic attractants, notably agrin and laminins, further strengthens axon–muscle connectivity and promotes accurate synapse formation [107]. These insights reposition muscle tissue from a passive recipient to a dynamic signaling hub that supports and directs nerve regeneration.

### Improvement of functional recovery and motor output

Ultimately, the success of any intervention for PNI hinges on its ability to restore motor function [14]. Skeletal muscle reprogramming improves functional outcomes by enhancing both structural and metabolic properties of the muscle. This approach upregulates key sarcomeric proteins, including myosin heavy chain and titin [108], which are essential for efficient force generation. It also counteracts denervation-induced fiber-type transitions by promoting a shift from fast glycolytic to oxidative phenotypes, thereby augmenting fatigue resistance and endurance [109]. Moreover, muscle reprogramming stabilizes the tendon–muscle interface and drives targeted ECM remodeling to restore effective force transmission across the myotendinous junction [110]. Functional benefits in rodent models—evidenced by enhanced EMG compound muscle action potentials, increased grip strength, and improved gait parameters—underscore the translational promise of these strategies [111].

### Synergy with nerve-centered therapies

Although skeletal muscle reprogramming is not intended to replace conventional nerve repair, it functions as a powerful adjunct when combined with nerve-centered therapies [112]. Nerve-centered therapies encompass a range of strategies designed to restore or reconstruct damaged nerves, including nerve grafting, neurotrophic scaffolds, and cell-based interventions, such as the transplantation of induced pluripotent stem cell (iPSC)-derived motor neurons [113, 114]. These interventions primarily aim to promote axonal regrowth, restore neural continuity, and reestablish functional synapses with target muscles. Despite their utility, the clinical success of these therapies is often limited by challenges such as poor receptivity of target muscles, misdirected axonal growth, and insufficient trophic or structural support for regenerating neurons, particularly in cases involving long-gap peripheral nerve injuries or delayed surgical repair [115]. Integrating skeletal muscle-targeted reprogramming with these nerve-centered modalities can overcome many of these limitations, yielding regenerative outcomes superior to those achieved by either approach alone. For example, simultaneous nerve grafting and localized delivery of muscle reprogramming factors not only enhances the intrinsi regenerative capacity of grafts but also improves axonal targeting and synaptic specificity within reinnervated muscles [116]. By optimizing the distal muscle environment, reprogramming strategies facilitate the precise alignment of regenerating axons with neuromuscular junctions, thereby promoting stable and functional connectivity [117]. Additionally, co-delivery of reprogramming factors with iPSC-derived motor neurons has been shown to establish a supportive regenerative niche, enriched with trophic cues and extracellular matrix components, which promotes neuronal survival, integration, and synapse formation [118]. Beyond cellular co-therapies, bioengineered scaffolds seeded with reprogrammed muscle cells can serve as hybrid conduits that bridge the muscle–nerve interface, offering both physical alignment for axonal growth and biochemical conditioning of the muscle to sustain neural input. These constructs enable synchronized regeneration of nerve and muscle tissues, addressing one of the major barriers to full functional recovery [119]. Such integrated approaches hold particular promise for long-gap injuries and delayed repair contexts, where temporal and spatial coordination of proximal axonal outgrowth with distal muscle preservation is critical. By simultaneously supporting nerve regeneration and maintaining a receptive, functional muscle substrate, skeletal muscle reprogramming amplifies the therapeutic impact of nerve-centered interventions, ultimately driving more robust and durable functional recovery [120].

### Clinical considerations and translational outlook

Despite encouraging preclinical outcomes, the translation of skeletal muscle reprogramming into clinical therapies faces multiple challenges [10]. Foremost are regulatory considerations for gene-modification and cell-transplantation approaches, including vector safety, manufacturing consistency, and long-term follow-up requirements [121]. Scaling protocols from rodent models to human patients necessitates optimizing dosage, delivery volumes, and accounting for slower regeneration kinetics and complex biomechanics in larger muscles [122]. Efficient, targeted delivery of reprogramming agents remains a pivotal hurdle, driving the development of minimally invasive modalities such as ultrasound-mediated injection and nanoparticle carriers [123]. Immune compatibility, particularly with viral vectors or allogeneic cells, further complicates clinical deployment and underscores the need for non-immunogenic delivery platforms [124]. Looking ahead, advances in biomaterials design, synthetic biology, and non-viral gene-delivery vectors hold promise to overcome these barriers. Rigorous clinical trials will be essential to evaluate the safety, efficacy, and durability of reprogramming-based interventions in diverse PNI patient populations.

## Recent advances in reprogramming strategies

In recent years, muscle reprogramming has evolved from proof-of-concept studies using transcription factors or small molecules to sophisticated, translationally oriented platforms [125]. These strategies not only boost regenerative capacity but also tackle critical obstacles—off-target effects, immune responses, and scalability—that have impeded clinical translation. Below, we highlight the most impactful innovations in skeletal muscle reprogramming for peripheral nerve injury repair, respective translational bottlenecks provided in Table 2.

**TABLE 2:** Recent advances in skeletal muscle reprogramming strategies for peripheral nerve injury repair.

Strategy/Advance	Clinical application value	Translational bottlenecks	References
Single-cell omics	Precise target discovery (e.g., distinguishing FAP subsets) and personalized therapy	High data complexity and significant inter-patient heterogeneity	[132, 133]
CRISPRa platforms Es	Endogenous gene reactivation (MyoD, Pax7) without permanent genetic modification. V	Viral vector capacity limits (e.g., AAV) and risk of off-target effects	[135, 173]
3D organoids/Bio-printing Hg	High-fidelity in vitro testing of synaptogenesis and neuromuscular unit maturation	Lack of complete vascularization and immune niche in engineered constructs	[110, 166]
AAV/Lipid nanoparticles Ts	Tissue-specific, non-invasive delivery of reprogramming tools to deep muscles	Potential immunogenicity of viral capsids and need for repeat dosing [g]	[125, 136]
Large animal models (Porcine/Canine) V)	Validation of long-gap nerve repair and human-scale biomechanics. H	High ethical/financial costs and slower regeneration kinetics compared to rodents	[138, 167]
Safety & ethics Es	Ensuring long-term functional stability and patient safety	Risks of oncogenic transformation, immune rejection of vectors, and epigenetic instability	[139, 140]

This table summarizes recent technological breakthroughs in the field and explores the translational bottlenecks from animal models to human clinical applications, directly addressing concerns regarding translational challenges. Single-cell multi-omics provides a precise blueprint for target discovery but is constrained by data complexity and inter-patient heterogeneity. CRISPR-based activation platforms allow for the precise reactivation of endogenous genes, yet they face hurdles related to vector capacity and potential immunogenicity. 3D organoids and bioprinting enable the *in vitro* reconstruction of functional neuromuscular units, although these models currently lack comprehensive vascular and immune system integration. Large animal models validate repair efficacy at a human-relevant scale, but they are characterized by slower regeneration kinetics and significantly higher ethical and financial costs.

### Single-cell multi-omics for target discovery

Recent single-cell RNA sequencing (scRNA-seq) and single-nucleus ATAC sequencing (snATAC-seq) studies have revealed extensive cellular heterogeneity and identified reprogramming-responsive subpopulations in injured muscle [126]. Fate-mapping analyses distinguish regenerative FAP subsets from those driving fibrosis, while integrated transcriptome–accessibility profiling uncovers master regulators of myogenic reprogramming [127]. Crucially, patient-derived single-cell signatures can guide personalized small-molecule cocktails or CRISPR targets by pinpointing dominant fibrotic or senescent pathways in individual samples.

### Small-molecule reprogramming cocktails

Defined small-molecule combinations now enable genetic-free induction of myogenic progenitors. For example, the “chemical iMPC” cocktail—CHIR99021 (GSK-3β inhibitor), forskolin (adenylate cyclase activator), and RepSox (TGF-β inhibitor)— efficiently converts mouse fibroblasts into induced myogenic progenitor cells capable of myotube formation and muscle engraftment [128]. Concurrent application of chromatin-modifying agents, such as trichostatin A (HDAC inhibitor) and JQ1 (BET inhibitor), further enhances accessibility at myogenic loci. Inhibition ofTGF-β and ROCK signaling reduces fibrosis and skews aged or chronically denervated tissues toward a regenerative phenotype. Because these molecules are reversible, titratable, and compatible with injectable hydrogels or nanoparticles, they represent a highly translatable approach.

### CRISPR-based epigenetic and transcriptional engineering

CRISPR/Cas systems now enable not only gene knockout but also precise activation (CRISPRa) or repression (CRISPRi) of endogenous myogenic regulators. Activation platforms—dCas9-VP64 and dCas9-p300—have been applied to upregulate MyoD, Pax7, and Myf5 in fibroblasts and FAPs, driving high-fidelity myogenic conversion without introducing exogenous sequences [129]. In contrast, CRISPRi targeting of anti-myogenic factors (e.g., Pparg, Tgfbr1) attenuates fibrotic signaling and shifts the stromal niche toward regeneration. Emerging epigenome editors, which fuse dCas9 to histone methyltransferases or demethylases such as LSD1 and TET1, permit locus-specific chromatin remodeling, thereby affording spatially and temporally precise lineage control in complex neuromuscular environments.

### 3D culture systems and organoids

Three-dimensional culture platforms better recapitulate native muscle biomechanics and architecture than traditional 2D systems [105]. Myospheres and myobundles—engineered from reprogrammed progenitors—exhibit contractile activity and sustain functional neuromuscular junctions *in vitro*. Neuromuscular organoids, generated by co-culturing these constructs with motor neurons, enable dynamic studies of synaptogenesis and reinnervation. Furthermore, 3D bioprinting of cell-laden bioinks produces grafts tailored to complex nerve-injury geometries, facilitating high-throughput optimization of reprogramming parameters prior to *in vivo* application.

### *In vivo* gene delivery systems

Effective clinical translation requires tissue-specific, efficient delivery of reprogramming tools. Adeno-associated viruses—especially serotypes AAV9 and AAVrh74—have successfully delivered MyoD or CRISPRa components directly into skeletal muscle [130]. As a non-viral alternative, lipid nanoparticles carrying mRNA or small molecules offer minimal immunogenicity and enable repeat dosing. Injectable hydrogel scaffolds, responsive to muscle-specific enzymatic cues, further improve local retention and controlled release, collectively mitigating off-target expression and systemic toxicity.

### Preclinical and early clinical studies

Most reprogramming interventions remain at the preclinical stage, yet murine sciatic-nerve transection models demonstrate restored electromyographic activity, increased muscle force, and enhanced NMJ density [131]. Large-animal studies in canine and porcine models are underway to evaluate reprogrammed muscle grafts within nerve conduits [132]. Early-phase human trials—initially targeting Duchenne muscular dystrophy—have established the safety and feasibility of AAV-mediated MyoD or IGF-1 delivery, providing valuable insights for PNI applications.

### Ethical and safety considerations

As muscle reprogramming advances toward the clinic, robust safety frameworks are essential. Key risks include oncogenic transformation of proliferative progenitors, immune rejection of viral vectors or allogeneic cells, and epigenetic instability that may compromise long-term function [133]. Future efforts should standardize manufacturing protocols, integrate reversible “kill-switch” mechanisms, and implement extended monitoring to ensure clinical safety and efficacy [134].

## Complications addressed through muscle reprogramming

Peripheral nerve injury (PNI) not only disrupts axonal continuity but also triggers a cascade of maladaptive changes within skeletal muscle and the broader neuromuscular unit. Denervated muscle develops fibrosis, fatty infiltration, chronic pain, neuromuscular junction (NMJ) degradation, and satellite cell exhaustion, culminating in functional failure despite technically successful nerve repair. By reprogramming muscle cells and their microenvironment, pathological phenotypes can be remodeled and pro-regenerative signaling networks restored.

### Muscle fibrosis

Denervation drives excessive deposition of extracellular matrix proteins—mainly collagen I and III—that stiffen tissue, impair mechanotransduction, and reduce contractile efficiency. Reprogramming strategies redirect fibro-adipogenic progenitors away from fibrogenic differentiation via transient TGF-β inhibition and locus-specific epigenetic editing of profibrotic genes [8, 135]. Concurrent modulation of matrix metalloproteinases and their inhibitors rebalances ECM turnover, improves compliance, and facilitates regenerating axons’ penetration into the muscle scaffold.

### Fatty infiltration and adipogenic conversion

Chronic denervation skews fibro-adipogenic progenitors toward adipocyte formation, leading to lipid accumulation that disrupts contractile architecture. Small-molecule reprogramming—using PPARγ antagonists with Wnt/β-catenin agonists—rebalances lineage choice in favor of myogenesis [96]. Activation ofthe IGF-1/Akt axis further suppresses adipogenic networks, promoting myofiber hypertrophy and metabolic resilience. In rodent models, this combinatorial approach reduced intramuscular fat by 60% and improved grip strength by 30% compared to controls [136].

### Neuropathic pain modulation

Persistent neuropathic pain following PNI arises from ectopic neural firing, central sensitization, and elevated proinflammatory cytokines in denervated muscle. Muscle-centric reprogramming that shifts cytokine milieus—decreasing IL-6 and TNF-α while boosting IL-10—has attenuated mechanical allodynia in sciatic-transected rats [137]. Restoration of NMJ integrity through agrin mimetics further normalizes aberrant afferent signaling and reduces dorsal-horn hyperexcitability.

### NMJ disassembly and synaptic drift

Denervation disrupts the agrin–MuSK–LRP4 signaling axis, dispersing acetylcholine receptor clusters and retracting presynaptic terminals. Epigenetic upregulation of Rapsyn and Dok-7 in reprogrammed myofibers enhances AChR clustering and stabilizes terminal Schwann cell processes, preserving NMJ architecture during prolonged denervation [138]. This extended window enables more effective nerve reconnection and improves post-repair synaptic transmission.

### Satellite cell senescence and exhaustion

**S**ustained denervation drives satellite cells into senescence, depleting the myogenic progenitor pool. Targeted CRISPRa activation of Pax7 and MyoD revives quiescent MuSCs and reinstates youthful chromatin landscapes—evidenced by telomere elongation and reduced β-galactosidase activity [139]. Concurrent modulation of the niche with anti-inflammatory cytokines creates a supportive microenvironment that sustains long-term progenitor function.

### Regenerative failure and non-responsive denervation

When both nerve and muscle regeneration fail, inducible reservoirs of myogenic cells can be generated from interstitial stromal populations. Secretion of neurotrophic factors such as GDNF and BDNF by reprogrammed muscle grafts attracts axonal sprouts and forms functional neuromuscular interfaces when combined with exogenous stem cell therapies [140]. In porcine models, these hybrid constructs bridge critical-sized nerve gaps and recover up to 70% of baseline muscle force.

### Vascular remodeling and immunomodulation

Effective reinnervation also depends on adequate vascular support and immune balance. Co-delivery of VEGF with myogenic reprogramming factors enhances angiogenesis in denervated muscle, improving nutrient delivery and waste clearance [141]. Simultaneous polarization of macrophages toward an M2 phenotype reduces chronic inflammation and provides additional pro-regenerative cytokines, further facilitating axonal regrowth. By integrating these multifaceted muscle reprogramming strategies—targeting fibrosis, adipogenesis, pain, NMJ integrity, satellite cell rejuvenation, regenerative salvage, vascular support, and immune modulation—we can comprehensively address PNI- induced complications and extend the therapeutic window for successful nerve repair.

## Discussion

### Challenges and limitation

Despite encouraging preclinical outcomes, translating skeletal muscle reprogramming for peripheral nerve injury (PNI) into clinical therapies remains hindered by biological constraints, delivery inefficiencies, safety concerns, and complex regulatory and economic landscapes [142]. Overcoming these barriers is essential to move reprogramming approaches from laboratory prototypes into standardized treatments. The injured muscle niche contains satellite cells, fibro-adipogenic progenitors (FAPs), endothelial cells, immune infiltrates, and resident fibroblasts—any of which may inadvertently respond to reprogramming cues. Unintended modulation of non-myogenic populations risks aberrant remodeling and inflammation [143]. Moreover, most vectors use ubiquitous promoters lacking cell-type specificity. Recent strategies employ microRNA-regulated expression cassettes that remain inert in off-target cells but activate transgenes selectively in FAPs or myoblasts [144].

Converting stromal cells into early myogenic progenitors does not guarantee their maturation into fully contractile fibers. A significant fraction of induced cells stall at the MyoD-positive stage and fail to upregulate late structural genes such as myosin heavy chain [145]. Even when new fibers form, misalignment with regenerating axons can produce unstable or non-functional neuromuscular junctions. Beyond structural assembly, a significant “translational value gap” exists; traditional functional assessments in rodent PNI models, such as simple gait analysis, often fail to capture the complex neuromuscular coordination and fine motor control essential for human clinical recovery [146]. Engineering biomaterial scaffolds with aligned topography guides both myofiber orientation and axonal trajectories, improving integration.

Reprogramming—especially using viral vectors, synthetic mRNAs, or chromatin modifiers—can provoke innate and adaptive immunity. Repeat AAV administrations frequently elicit neutralizing antibodies, reducing transduction efficacy in large-animal and human studies. Off-target epigenetic remodeling may activate cGAS–STING signaling, triggering proinflammatory cascades that impair regeneration [147].

Immunomodulatory co-therapies, such as transient corticosteroids or localized macrophage reprogramming, are under investigation to mitigate these responses.

Reprogramming protocols often transiently suppress tumor suppressors (e.g., p53) to enhance plasticity, inadvertently increasing genomic instability. Sustained overexpression of myogenic transcription factors has been linked to rhabdomyosarcoma-like lesions in rodent models [148]. To reduce oncogenic risk, inducible “suicide switch” constructs—such as drug-activated caspase-9 systems—allow selective ablation of aberrantly proliferating cells [149]. While previous studies focused on macroscopic tumor formation, a more critical analytical framework is now required to monitor dynamic genomic stability. Recent 2025 clinical evaluations of human iPSC technologies emphasize that establishing rigorous standards for genomic integrity is a prerequisite for any clinical transition of reprogramming therapies [150]. The efficacy of reprogramming diminishes as denervation becomes chronic: satellite cells senesce, ECM cross-links accumulate, and synaptic basal lamina degrades [68]. Experimental data indicate a critical therapeutic window of approximately two to 4 weeks post-denervation for optimal responsiveness [151]. Early injury detection via advanced imaging or biomarker panels is therefore crucial to enable timely intervention. Scaling delivery from rodents to humans introduces logistical challenges: achieving uniform vector or small-molecule distribution across large, deep muscle compartments is difficult, and repeated intramuscular injections are impractical [152]. This “translational disconnect” is exacerbated by the vast anatomical differences between species; human muscles present severe physical diffusion barriers and varying tissue densities that prevent the uniform distribution of reprogramming factors, a challenge often oversimplified in standardized laboratory settings [125]. Non-invasive methods—such as focused ultrasound–mediated vascular delivery—transiently increase vascular permeability, enabling systemic agents to penetrate target tissues [153].

Reprogramming therapies often combine gene editing, drug delivery, and celltransplantation, falling into ambiguous regulatory categories that vary by region [154]. Ethical concerns over germline editing and off-target CRISPR effects continue to provoke public debate. Furthermore, while small-molecule “molecular time machines” offer a promising route to reverse senescence without genetic modification, their potential systemic side effects and disruption of long-term genomic stability in humans necessitate rigorous critical scrutiny beyond initial experimental success [155]. Additionally, the high cost of GMP-grade vectors, personalized cell products, and prolonged rehabilitation regimens presents an economic burden that may limit patient access [156].

### Future directions and research gaps

**T**he field of skeletal muscle reprogramming in the context of peripheral nerve injury (PNI) has achieved significant milestones, yet key questions remain unanswered. As efforts shift toward clinical translation, it is imperative to identify synergistic strategies, resolve outstanding biological uncertainties, and adopt standardized protocols. The followingsections outline six priority areas where concerted research can accelerate therapeutic innovation.

The integration of muscle and nerve reprogramming is crucial for achieving functional recovery after PNI, as current therapies often target these systems in isolation. Recent studies suggest that coordinated repair is essential, with evidence indicating that reprogramming skeletal muscle can enhance its ability to accept innervation. For instance, a 2024 study by Mehrotra et al. demonstrated that transient expression of NANOG in skeletal muscle improves neuromuscular junction formation and functional recovery in murine models of sciatic nerve injury [10]. This approach upregulates genes involved in muscle development and axon guidance, supporting synchronized regeneration. Additionally, *in vitro* models like neuromuscular organoids offer insights into the molecular interactions between muscle and nerve cells. A 2021 study in *Nature Communications* developed human sensorimotor organoids from induced pluripotent stem cells (iPSCs), revealing physiologically functional neuromuscular junctions and providing a platform to study disease mechanisms [157]. These models highlight retrograde signaling loops, such as those involving glial cell line-derived neurotrophic factor (GDNF), which may promote Schwann cell maturation. Advanced techniques like single-cell multi-omics and spatial transcriptomics are increasingly applied to delineate bidirectional molecular dialogues, though specific studies directly linking these to PNI are still emerging.

Achieving high-fidelity reprogramming requires tools that operate selectively in target cells and adapt to changing tissue states, minimizing off-target effects. Research suggests that pH-responsive hydrogels can release reprogramming factors in the acidic microenvironment of denervated muscle, enhancing specificity. A review in PMC highlighted the potential of pH-sensitive hydrogels for drug delivery, which could be adapted for muscle reprogramming [158]. This approach reduces unintended effects on healthy tissue, aligning with the need for context-sensitive strategies. MicroRNA-based regulation is another promising avenue, with microRNA-206 (miR-206) playing a significant role in muscle differentiation and regeneration. Studies show that miR-206 promotes myoblast differentiation and regulates satellite cell proliferation by repressing Pax7, confining transgene expression to myogenic progenitors [105]. Timing the delivery of epigenetic modulators to match tissue repair phases, such as the transition from inflammation to proliferation, is also critical, with evidence suggesting improved efficiency and functional integration, though specific studies on PNI are limited.

Robust *in vitro* platforms that recapitulate neuromuscular complexity are essential for preclinical validation, reducing reliance on animal models. Recent advances in 3D bioprinting have facilitated the creation of muscle-nerve constructs, incorporating aligned extracellular matrix (ECM) fibers and reprogrammed progenitors. A study demonstrated that neural cell integration into 3D bioprinted skeletal muscle constructs accelerates functional muscle regeneration *in vivo*, allowing precise assessment of fiber orientation and axonal targeting [159]. These constructs can be enhanced with optogenetic stimulation and high-resolution calcium imaging for real-time quantification of contractile kinetics and synaptic transmission, standard techniques in neuroscience research. To improve translational relevance, microfluidic “body on a chip” devices using human iPSC-derived fibro-adipogenic progenitors and motor neurons are being explored. A 2023 study in *eLife* developed human skeletal muscle organoids that sustain uncommitted Pax7-positive myogenic progenitors, offering a model for personalized assessment of reprogramming cocktails [160]. These platforms pave the way for tailoring therapies to individual genetic backgrounds, though challenges remain in scaling and standardization.

Translating findings from rodents to larger, anatomically and immunologically comparable species is a critical step for clinical application. Studies in large animal models, such as canines and porcines, have shown the feasibility of AAV-mediated gene delivery for muscle-related disorders. For example, a 2011 study in PMC demonstrated whole-body skeletal muscle transduction in neonatal dogs using AAV-9, leading to functional improvements in muscle strength [161]. While specific studies on PNI using muscle reprogramming in large animals are limited, the general approach of gene therapy for nerve regeneration is actively explored, with evidence suggesting potential for restoring gait and muscle fiber cross-sectional area. Human trials for gene therapy in PNI are still in early stages, with no widely reported clinical trials identified in recent literature.

Long-term outcomes and safety profiling are essential for clinical translation, yet longitudinal data remain scarce. Preliminary studies in rodent models suggest that reprogrammed muscles can maintain stable transgene expression without evidence of tumorigenesis. A 2024 study in *Nature Communications* on skeletal muscle reprogramming for PNI monitored outcomes over extended periods, showing preserved contractile strength under chronic exercise, though specific long-term safety data are limited [10]. Lineage tracing studies, a standard approach in gene therapy, have been used to ensure no clonal expansions or aberrant growths, supporting the safety of epigenetic reprogramming approaches, but further research in larger models is needed. Functional assessments, such as subjecting reprogrammed muscles to mechanical stress, indicate resilience, with a 2022 meta-analysis in *Journal ofScience in Sport and Exercise* suggesting long-lasting stretching induces muscle hypertrophy, relevant for maintaining function [162]. However, the field requires more comprehensive data to address safety concerns, particularly in humans, to ensure long-term efficacy and minimize risks like immune responses or unintended cellular changes.

Breakthroughs will emerge at the interface of biology, engineering, and computation. Park et al. (2023) showed that cyclic mechanical stretch, applied via bespoke bioreactors, enhances MyoD-mediated reprogramming efficiency by over 30 percent through mechanotransductive activation of YAP signaling [163]. Wang et al. (2021) developed *in silico* models that integrate diffusion dynamics of reprogramming factors with cellular signaling networks, accurately predicting optimal dosing schedules later confirmed *in vivo* [164]. To foster collaboration and reproducibility, Thompson et al. (2024) launched the open‐access NeuroMuscle Atlas, a repository cataloging reprogramming outcomes, vector designs, and safety metrics from multiple laboratories. By pursuing integrated muscle–nerve reprogramming, refining cell-specific tools, engineering advanced *in vitro* platforms, validating in large mammals, establishing long-term safety, and embracing interdisciplinary approaches, the field can transition muscle reprogramming from experimental promise to clinical reality.

## Conclusion

Peripheral nerve injury (PNI) remains a formidable clinical challenge because of its biological complexity, high incidence, and frequent progression to irreversible functional deficits. Despite notable advancements in microsurgical repair techniques, including refined fascicular alignment and tension-minimized nerve coaptation [165], as well as adjunctive strategies such as neurotrophic factor delivery and bioengineered conduits, these interventions primarily address nerve regeneration while insufficiently mitigating the denervated muscle environment [166, 167]. Without targeted intervention, denervated skeletal muscle rapidly undergoes atrophy, fibrosis, and neuromuscular junction (NMJ) degradation, which severely limits the success of nerve repair.

To address this gap, skeletal muscle reprogramming—defined as the deliberate modification of muscle-resident or stromal cell fate via coordinated transcriptional, epigenetic, and metabolic interventions—has emerged as a promising therapeutic avenue. Preclinical studies demonstrate that reprogramming can preserve muscle mass during prolonged denervation, reverse fibrotic and adipogenic remodeling, and promote NMJ reformation via retrograde signaling loops, collectively sustaining muscle receptivity to reinnervation [10]. Notably, combinatorial approaches integrating reprogrammed muscle with nerve-centered strategies (e.g., nerve grafts or neurotrophic scaffolds) yield synergistic improvements in functional recovery compared with either modality alone [116].

Mechanistically, these benefits result from the coordinated activity of myogenic transcription factors (e.g., MyoD, Pax7), chromatin modifiers (HDACs, EZH2), metabolic sensors (AMPK, SIRT1), and paracrine mediators such as IGF-1 and agrin [168]. Recent technological advances—including CRISPR-based activation platforms for endogenous myogenic genes, defined small-molecule cocktails that promote progenitor expansion and reduce fibrosis, 3D neuromuscular co-culture systems that recapitulate functional NMJs *in vitro*, and targeted delivery vehicles such as adeno-associated viruses (AAVs) and biodegradable nanoparticles—have collectively propelled the field toward clinical translation [169–171].

Nevertheless, significant challenges remain. Cell specificity is hindered by off-target transduction and heterogeneous tissue responses, while long-term safety concerns include the risk of tumorigenesis from proliferative progenitors [172]. Moreover, most efficacy data are derived from rodent models, necessitating validation in large animals and eventual early-phase clinical trials under evolving regulatory oversight [173, 174]. To ensure reproducibility and equitable patient access, standardized potency assays and GMP- compliant manufacturing protocols must also be established.

Looking ahead, we recommend the creation of multicenter consortia to develop consensus guidelines for *in vivo* muscle reprogramming, including standardized injury models, dosing regimens, and outcome metrics. Simultaneously, the refinement of non-invasive monitoring tools—such as advanced magnetic resonance imaging (MRI) and circulating biomarkers—will be critical to track reprogramming dynamics and NMJ integrity in real time. The integration of computational modeling and machine learning can further optimize intervention timing and predict patient-specific therapeutic responses, accelerating personalized clinical translation [175].

In conclusion, skeletal muscle reprogramming represents a paradigm shift in PNI management, transforming denervated muscle from a passive bystander into an active driver of regeneration. By targeting the pathological muscle niche in concert with nerve repair, these strategies offer the potential for more complete and durable functional restoration. Realizing this promise will require interdisciplinary collaboration spanning molecular biology, bioengineering, clinical surgery, and regulatory science to translate compelling preclinical findings into safe and effective therapies for patients suffering from peripheral nerve injuries.
